# Kallikrein-related peptidases 6 and 10 are elevated in cerebrospinal fluid of patients with Alzheimer’s disease and associated with CSF-TAU and FDG-PET

**DOI:** 10.1186/s40035-019-0168-6

**Published:** 2019-08-27

**Authors:** Oliver Goldhardt, Inanna Warnhoff, Igor Yakushev, Ilijana Begcevic, Hans Förstl, Viktor Magdolen, Antoninus Soosaipillai, Eleftherios Diamandis, Panagiotis Alexopoulos, Timo Grimmer

**Affiliations:** 1 Department of Psychiatry and Psychotherapy, Klinikum rechts der Isar, Technical University of Munich, School of Medicine, Ismaninger Str. 22, 81675 Munich, Germany; 2 Department of Nuclear Medicine, TUM-NIC, Klinikum rechts der Isar, Technical University of Munich, School of Medicine, Ismaninger Str. 22, 81675 Munich, Germany; 3 Department of Obstetrics & Gynecology, Klinikum rechts der Isar, Technical University of Munich, School of Medicine, Ismaninger Str. 22, 81675 Munich, Germany; 4 Department of Pathology and Laboratory Medicine, Mount Sinai Hospital, 60 Murray St., Toronto, Ontario M5T 3L9 Canada; 5 Department of Laboratory Medicine and Pathobiology, Faculty of Medicine, University of Toronto, Medical Science Building, 1 King’s College Circle, Toronto, Ontario M5S 1A8 Canada; 6 Department of Psychiatry, University hospital of Rion, University of Patras, 26500 Rion Patras, Patras, Greece

**Keywords:** Alzheimer’s disease (AD), Kallikrein-like peptidase (KLK), KLK6, KLK8, KLK10, Cerebral amyloid load, Cerebrospinal fluid (CSF), Amyloid 1–42; Aβ1–42; Aβ_42_, Tau protein, Total tau, tTau, Phospho tau, pTau, Positron emission tomography (PET)

## Abstract

**Background:**

Alterations in the expression of human kallikrein-related peptidases (KLKs) have been described in patients with Alzheimer’s disease (AD). We elucidated the suitability of KLK6, KLK8 and KLK10 to distinguish AD from NC and explored associations with established AD biomarkers.

**Methods:**

KLK levels in cerebrospinal fluid (CSF), as determined by ELISA, were compared between 32 AD patients stratified to A/T/(N) system with evidence for amyloid pathology and of 23 normal controls with normal AD biomarkers. Associations between KLK levels and clinical severity, CSF and positron emission tomography (PET) based AD biomarkers were tested for.

**Results:**

Levels of KLK6 and KLK10 were significantly increased in AD. KLK6 differed significantly between AD A+/T+/N+ and AD A+/T−/N+ or NC with an AUC of 0.922. CSF pTau and tTau levels were significantly associated with KLK6 in AD.

**Conclusions:**

KLK6 deserves further investigations as a potential biomarker of Tau pathology in AD.

**Electronic supplementary material:**

The online version of this article (10.1186/s40035-019-0168-6) contains supplementary material, which is available to authorized users.

## Background

In sporadic Alzheimer’s disease (AD) impaired cerebral amyloid clearance is considered to drive amyloid (Aβ) accumulation [1] which results in pathognomonic Aβ plaque formation [2]. Aβ initiates a pathophysiological cascade including neuronal injury. Accumulative neuronal injury can be indicated by AD imaging biomarkers such as hypometabolism by [^18^F]fluorodeoxyglucose-position emission tomography ([^18^F]FDG-PET) [3]. Current rate of cell death can be determined by elevated levels of the intracellular, microtubule originated total tau (tTau) and tau pathology by phosphorylated tau (pTau) in the cerebrospinal fluid (CSF) [4]. Aβ accumulation can be measured by [^11^C]PiB (Pittsburgh Compound B)-PET ([C^11^]PiB-PET) [5] and impaired amyloid clearance by decreased concentration of Aβ_42_ in the CSF [4].

Kallikrein-related peptidases (KLKs) constitute a family of 15 highly conserved trypsin- or chymotrypsin-like serine proteases [6] displaying various functions. All KLK genes are located on chromosome 19q13.4, in a region that has been associated with familiar AD [7]. KLK1, 4–8, 10, 11, 13 and 14 are expressed in the cortex as well as in the hippocampus [8, 9]. KLKs might also play a role in the pathophysiology of sporadic AD [10].

### KLK6

KLK6 was detected outside the brain in the serum, nipple aspirate fluid, breast cyst fluid, seminal plasma, amniotic fluid and breast cancer cytosols [11]. However, the highest expression of KLK6 in humans is observed in the central nervous system [8]. KLK6 is strongly expressed in the luminal cells lining the choroid plexus [12], in the grey matter of the brain and in peripheral nerves [13]. KLK6 is predominantly expressed in oligodendrocytes, pyramidal cells and astrocytes. It can be found in certain neuroendocrine cells [14] and in the endothelial cells of cerebral vessels. The latter suggests its involvement in the blood-brain barrier (BBB) [15]. KLK6 can cleave components of the BBB and can induce inflammation [16]. In non-injured CNS, KLK6 immunoreactivity is mainly found in oligodendroglia and neurons [17], while KLK6 is induced in astrocytes with neuronal injury [18]. In neurons, it is released from mitochondria into the cytoplasm due to various cellular stress and processes alpha-synuclein in Lewy-body disease [19].

KLK6 appears to exhibit an anti-amyloidogenic potential [20] and has been linked to AD: in extracts of different brain regions of patients with AD, KLK6 levels are lower as compared to controls [21, 22], KLK6 mRNA levels are decreased in the hippocampus as well [9]. In blood, current results are conflicting: one study did not detect any significant difference in KLK6 levels in the serum of AD patients as compared to cognitively normal control (NC) [23], whereas in another study a striking 10-fold increase in the blood was reported [12]. In the cerebrospinal fluid (CSF), KLK6 levels were increased in histopathologically confirmed AD patients (*n* = 10) of undisclosed clinical severity compared to controls (*n* = 10) [12]. In another study, CSF KLK6 levels did not differ neither between subjective cognitive impairment (*n* = 43) and AD (*n* = 43), nor between clinically diagnosed normal controls (*n* = 58) and clinically diagnosed AD (*n* = 28) or clinically diagnosed progressive mild cognitive impairment (MCI) (*n* = 28) [24].

### KLK8

KLK8 is a synaptic, plasticity-modulating extracellular serine protease [21]. KLK8 cleaves the epinephrine receptor B2 (EPHB2) that is involved in mechanisms of neuronal plasticity [25]. Upregulation of EPHB2 leads to improved cognition [26] and less hyperphosphorylated tau in vitro and in hippocampus in human tau transgenic mice [27]. In the hippocampus of AD patients and mice transgenic for human amyloid precursor protein (hAPP), KLK8 is upregulated and EPHB2 decreased [28, 29]. In transgenic AD mice, KLK8 is increased in early disease stages (before measurable behavior abnormalities occur) as compared to wildtype mice [30]. Furthermore, in mice, inhibition of KLK8 with anti-KLK8-antibodies leads to enhanced structural neuroplasticity, improved cognition, reduced Aβ concentrations in the frontal cortex, improved amyloid clearance over the BBB via increased concentrations of the Aβ efflux transporter lipoprotein-receptor related protein 1 (LRP1), increased Aβ phagocytosis, and counteracts tau hyperphosphorylation via downstream effects on GSK3β [30]. The impact of KLK8 on the neurovascular function to transport Aβ over the BBB can be measured in the blood in mice [30] and potentially in the CSF.

These associations have not been shown in humans with AD, but KLK8 mRNA expression is increased in the mural and human hippocampus in comparison to NC [9], and KLK8 levels are increased in hippocampal brain extracts patients with AD and in the brain of healthy woman compared to men [31].

### KLK10

KLK10 is expressed by glandular epithelia, e. g. in the kidney and in the gastrointestinal tract, and by the choroid plexus epithelium, by peripheral nerves, and by certain neuroendocrine organs [32]. Whereas KLK10 RNA expression in the hippocampus did not differ significantly between AD patients and NC [9], in CSF in AD, the protein levels were found to be decreased in frontotemporal dementia (FTD) [33].

Inflammation appears to be a common factor for alterations of KLKs levels in AD, cancer and other diseases [34] as well as inflammation appears to be a pathogenetic factor among several neurological diseases [35].

In AD, KLKs can be hypothesized to either be directly involved in amyloid accumulation or indirectly in immunological processes caused by or leading to amyloid pathology. In the current work, we aimed at testing whether KLK6, KLK8 and KLK10 levels in CSF are able to differentiate between AD patients with positive AD biomarkers and NC, and whether KLKs are associated with the established AD biomarkers.

## Methods

### Patient recruitment, inclusion and exclusion criteria

Patients were selected in the Centre for Cognitive Disorders, an out-patient clinic of the Department of Psychiatry and Psychotherapy of the Technical University of Munich. Study participants had been referred for the evaluation of cognitive impairment by general practitioners, neurologists, psychiatrists or other institutions, and had undergone a standardized diagnostic procedure with a detailed somatic, neurologic and psychiatric examination, as well as extensive neuropsychological testing, including the Mini-Mental State Examination (MMSE) [36]. To estimate clinical severity, the Clinical Dementia Rating scale (CDR) [37] was assessed, and the global score (global CDR) and the quantitative score of CDR subcategories (CDR-sum of boxes, CDR-SOB) [38] were calculated. Patients had to score ≥ 0.5 on global CDR to be considered for inclusion into the study.

No suspected or diagnosed severe life limiting disease or malignancy, multiple sclerosis, or idiopathic parkinsonism was present in any patient. All patients underwent cranial magnetic resonance imaging (MRI) on a 1.5 T scanner in order to assess structural brain abnormalities and vascular pathology such as ischemia and microbleeds. Clinical diagnosis of early AD, namely mild cognitive impairment or dementia due to AD, was made in accordance with the standard diagnostic criteria at the time of the study [39, 40] distinguishing AD biomarkers for amyloid pathology and for neurodegeneration. Patients were selected aiming for normal distribution of clinical severity (CDR-SOB) and age. Pathological levels in at least one biomarker of both neuronal injury (CSF-pTau > 60 ng/l, CSF-tTau > 252 ng/l or an AD-like hypometabolism in FDG-PET) and amyloid pathology (either abnormal CSF amyloid levels of Aβ_42_ < 634 ng/l or a positive amyloid PET) were required. Cut-offs for CSF-Aβ_42_ and CSF-tTau were derived from previous publications [41]; cut-off for CSF-pTau was established in-house. Thirty-two patients were included. Thirty patients had pathological results in both categories. In two patients CSF amyloid levels were within normal range but converted to pathological levels at a two-year follow-up CSF analysis. Additionally, all CSF samples were assessed for cells, protein concentrations, including albumin serum to CSF ratios (R_alb_), oligoclonal bands, lactate and glucose.

Cognitively normal controls (NC, *n* = 23) did not report any memory deficits and scored within the normal range on psychometric testing using the MMSE [42]. All NCs scored 0 on the global CDR. Members of the control group did not have the suspected or confirmed diagnosis of cancer, MS, or parkinsonism. NCs were required to show normal values regarding CSF pTau (< 60 ng/l), Aβ_42_ (> 634 ng/l) and Aβ_42_/Aβ_40_ ratio (> 0.05, in house cut-off). All NCs showed normal CSF tTau levels (< 252 ng/l), except for one with a CSF tTau level of 266 ng/ml (within normal limits for pTau, Aβ_42_ and Aβ_42_/Aβ_40_).

All participants provided written informed consent. The local ethics committee approved the study protocol and all examinations were conducted in accordance with the Declaration of Helsinki, sixth revision.

Apolipoprotein E epsilon 4 allele frequency (ApoEε4) was determined in all but one participants [43]. All patients underwent cMRI to assess structural abnormalities, and vascular pathology such as ischemia and microbleeds.

### Brain imaging

In 14 AD patients with AD typical CSF markers, PET with [^18^F]FDG and [^11^C]PiB on the same cMRI/PET scanner were performed. Twelve of these patients showed an hypometabolism in the temporo-parietal and/or posterior cingulate cortex with relative sparing of the primary sensomotor cortex on visual inspection at [^18^F]FDG-PET [44] in accordance with AD typical findings [3, 45]. The two remaining patients exhibited regional unspecific hypometabolism. All 14 patients showed pathological cerebral [^11^C]PiB tracer uptake. PET analyses were performed using standard procedures [46–48]. Further details of the PET methods are provided in the Additional file 1.

### CAA and vascular pathology

To reduce the likelihood of either a co-existent cerebral amyloid angiopathy (CAA) or marked vascular pathology, patients were not allowed to show more than two lacunar infarcts, postischemic areas with more than 2 cm in diameter on fluid-attenuated inverse recovery (FLAIR) MRI-images, or > 4 microbleeds on T2* images [49].

### CSF ELISA (Aβ_42_, Aβ_40,_ total tau, phosphorylated tau)

CSF sampling and analyses have been described elsewhere [50]. CSF-Aβ_42_ and CSF-Aβ_40_ were measured in triplicate, total tau (CSF-tTau) and phosphorylated Tau (CSF-pTau) were measured in duplicate using commercially available enzyme-linked immunosorbent assays (ELISAs) (Innogenetics, Ghent, Belgium). The coefficients of variation (CVs) were less than 6% for CSF-Aβ_42_ and CSF-Aβ_40,_ and less than 3% for CSF-tTau and CSF-pTau).

### CSF ELISA (KLK6, KLK8, KLK10)

Immunofluorometric ELISA techniques have been described elsewhere [11, 51, 52]. KLKs were blinded before measured in duplicate. The inter-assay CVs were less than 10% for all assays [11, 51, 52]. All KLK-ELISA were consistent with the precision of typical microtiter plate-based immunoassays. For CSF, recovery percentages for all KLKs were between 90 and 110%. The antibody against KLK6 does not exhibit cross-reactivity with a series of different KLKs, KLK2, KLK3 (PSA), KLK8, KLK10, KLK11, KLK13 and KLK14, even when the cross-reactants were tested at concentrations of 1000 μg/L [22]. The antibody against KLK8 displayed no cross-reactivity against members of the human kallikrein family [52]. Finally, the antibody against KLK10 showed no cross-reactivity with other homologous kallikrein proteins, such as KLK3, KLK2, and KLK6, as well [51].

## Statistical analyses

### Primary analyses

#### Group differences

To test whether KLK6, KLK8 or KLK10 differ between AD and NC Mann-Whitney-U-tests were calculated. In case the variables were distributed normally (applying the Kolmogorow-Smirnow test), a t-test was performed. Receiver-operating characteristics (ROC) curves for the three KLKs with an area under the curve (AUC) were received, and from these ROC curves likelihood ratios for AD or for NC (LR_AD_ = sensitivity/(1-specificity) & LR_NC_ = (1-sensitivity)/specificity)) were calculated for best cut-offs. To account for age, sex and ApoE ε4 frequency imbalances in the whole sample, general linear models (ANOVA) of KLK6, KLK8 or KLK10 and diagnostic group (AD_y/n_) including possible confounders (age, sex, ApoE ɛ4 allele frequency) were calculated. The interaction terms AD_y/n_ x age, AD_y/n_ x sex and/ or AD_y/n_ x ApoEε4 frequency were included, if they explained additional variance. To examine whether KLKs were correlated with clinical severity measured by MMSE or global CDR, Spearman two-sided correlation analyses were calculated within the AD group.

### A/T/(N) system

The AD group was stratified to new research criteria (A/T/(N) system) [53, 54] and the AD subgroups A+/T+/N+, A + T-N+ and NC were compared.

### Associations of KLKs with AD biomarkers in CSF

To examine whether CSF KLK6, KLK8 or KLK10 concentrations are associated with the CSF biomarkers for AD (CSF-Aβ_42_, CSF-tTau, CSF-pTau), linear regression analyses with the dependent variable CSF-Aβ_42_ (or CSF-tTau or CSF-pTau, respectively) and the independent variable KLK6, KLK8 or KLK10, were performed separately in the patient sample and in NC. Covariates (age, sex, ApoE ɛ4 allele frequency, global CDR, MMSE) that explained variability (as measured by an increase of R^2^ adjusted) were included stepwise.

### Exploratory analyses

Linear regression models with the global FDG-PET or PiB-PET signal to reference region (cerebrum to cerebellum or to pons or to thalamus ratios) as dependent variables with KLK6, KLK8 or KLK10 as independent variables, respectively, were calculated in a sub-cohort of 14 AD patients.

In the subgroup of 14 AD patients with PiB and FDG PET data regional voxel-based PET analyses between KLKs and regional tracer uptake were performed (software: MathWorks® MATLAB R2016b, Natick, MA, USA; Statistical parametric mapping for positron emission tomography (SPM PET) 12, Wellcome Centre for human neuroimaging at University College London). Covariates (age, sex, ApoE ɛ4 allele frequency, global CDR, MMSE) that explained variability were included. The level of significance was set to 0.05 (family-wise error (FWE) corrected at cluster level. Negative and positive associations were examined.

To test whether CSF KLK6, KLK8 or KLK10 are associated with each other, linear regression analyses were calculated with each KLK as dependent variable and the other KLKs as independent variables separately within the AD and the NC group.

Additionally, plausibility analyses were performed to investigate whether KLK levels in the CSF are associated with an impaired BBB (Additional file 1).

## Results

### Primary analyses

#### Group differences

**Fig. 1:**
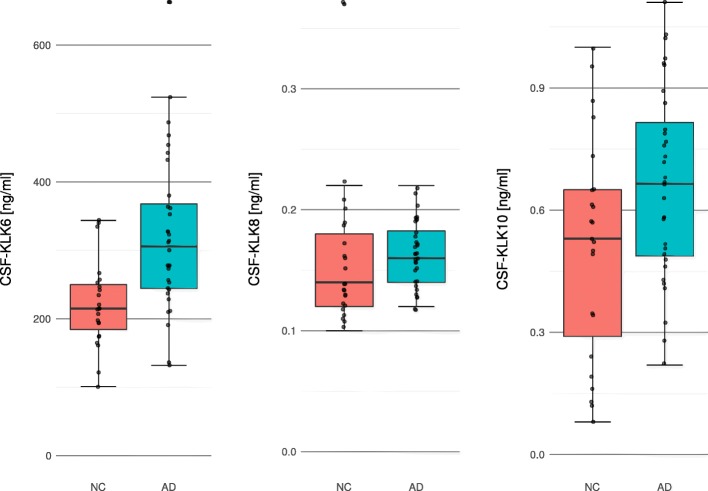
Distribution of KLK6, KLK8 and KLK10 in CSF of NC and AD. Between NC and AD, CSF-KLK6 (*p* < 0.001) and CSF-KLK10 (*p* = 0.008), but not KLK8 (*p* = 0.637), differed significantly when using t-tests. CSF: cerebrospinal fluid, NC: cognitively normal controls, AD: patients with Alzheimer’s disease, KLK: Kallikrein-related peptidase

KLK6, KLK8 and KLK10 were normally distributed in AD (*p*-values: p_KLK6_ = 0.156, p_KLK8_ = 0.200, p_KLK10_ = 0.200) and in NC (p-values: p_KLK6_ = 0.200, p_KLK8_ = 0.051, p_KLK10_ = 0.200). All KLK mean levels were numerically higher in AD as compared to NC. A box plot of individual measurement points of KLKs in the CSF between AD and NC is shown in Fig. 1. KLK6 and KLK10, but not KLK8, differentiated significantly between AD and NC when using t-tests (*p* < 0.001, 0.008 and 0.637, respectively). Participants’ characteristics are provided in Table 1.

**Table 1:** Characteristics of participants

Variables	AD *N* = 32	NC *N* = 23	*p* values
global CDR frequency 0/0.5/1/2/3	0 / 24 / 7 / 1 / 0	23 / 0 / 0 / 0 / 0	< 0.001
CDR-SOB mean ± SD (range)	3.5 ± 1.84 (0.5–9)	0.0 ± 0.00 (0.0–0.0)	< 0.001
MMSE score mean ± SD (range)	23.6 ± 4.37 (9–29) *n* = 31	29.5 ± 0.79 (28–30)	< 0.001
Sex male / female	14 / 18	18 / 5	0.011
Age in years mean ± SD (range)	71.5 ± 4.70 (61–82)	64.7 ± 9.16 (50–85)	0.002
ApoE ε4 allele frequency 0/1/2/n.a.	9 / 15 / 4 / 4	15 / 8 / 0	0.010
CSF-Aβ_42_ levels [ng/l] mean ± SD (range)	516 ± 145.6 (260–930)	1014 ± 199.0 (668–1354)	< 0.001*
Aβ_42_/Aβ_40_ ratios mean ± SD (range)	0.040 ± 0.0094 (0.020–0.061)	0.067 ± 0.0096 (0.051–0.0813)	< 0.001*
CSF t-tau levels [ng/l] mean ± SD (range)	588 ± 334 (166–1650)	182 ± 41.3 (108–266)	< 0.001*
CSF p-tau levels [ng/l] mean ± SD (range)	74.8 ± 32.5 (36–176)	35.5 ± 7.48 (23–53)	0.001*
CSF-KLK6 ng/ml mean ± SD (range)	321 ± 115.8 (132–663)	220 ± 62.4 (101–344)	< 0.001*
CSF-KLK8 ng/ml mean ± SD (range)	0.163 ± 0.026 (0.12–0.22)	0.157 ± 0.0580 (0.10–0.37)	0.637*
CSF-KLK10 ng/ml mean ± SD (range)	0.665 ± 0.2338 (0.22–1.11)	0.484 ± 0.0249 (0.08–0.95)	0.008*

*p* values: results of Mann-Whitney-U test or (*) t-test, respectively. In five patients, CDR was rated due to doctor’s letter, and one patient underwent the Montreal Cognitive Assessment test (MOCA test) instead of the MMSE

*AD* patients with Alzheimer’s disease, *NC* cognitively normal controls, *SD* standard deviation, *CDR* clinical dementia rating scale, *SOB* sum of boxes, *GLO* global, *MMSE* Mini-Mental State Examination, *ApoE* Apolipoprotein E, *CSF* cerebrospinal fluid, *Aβ* β-amyloid, *KLK* kallikrein related peptidase

**Fig. 2:**
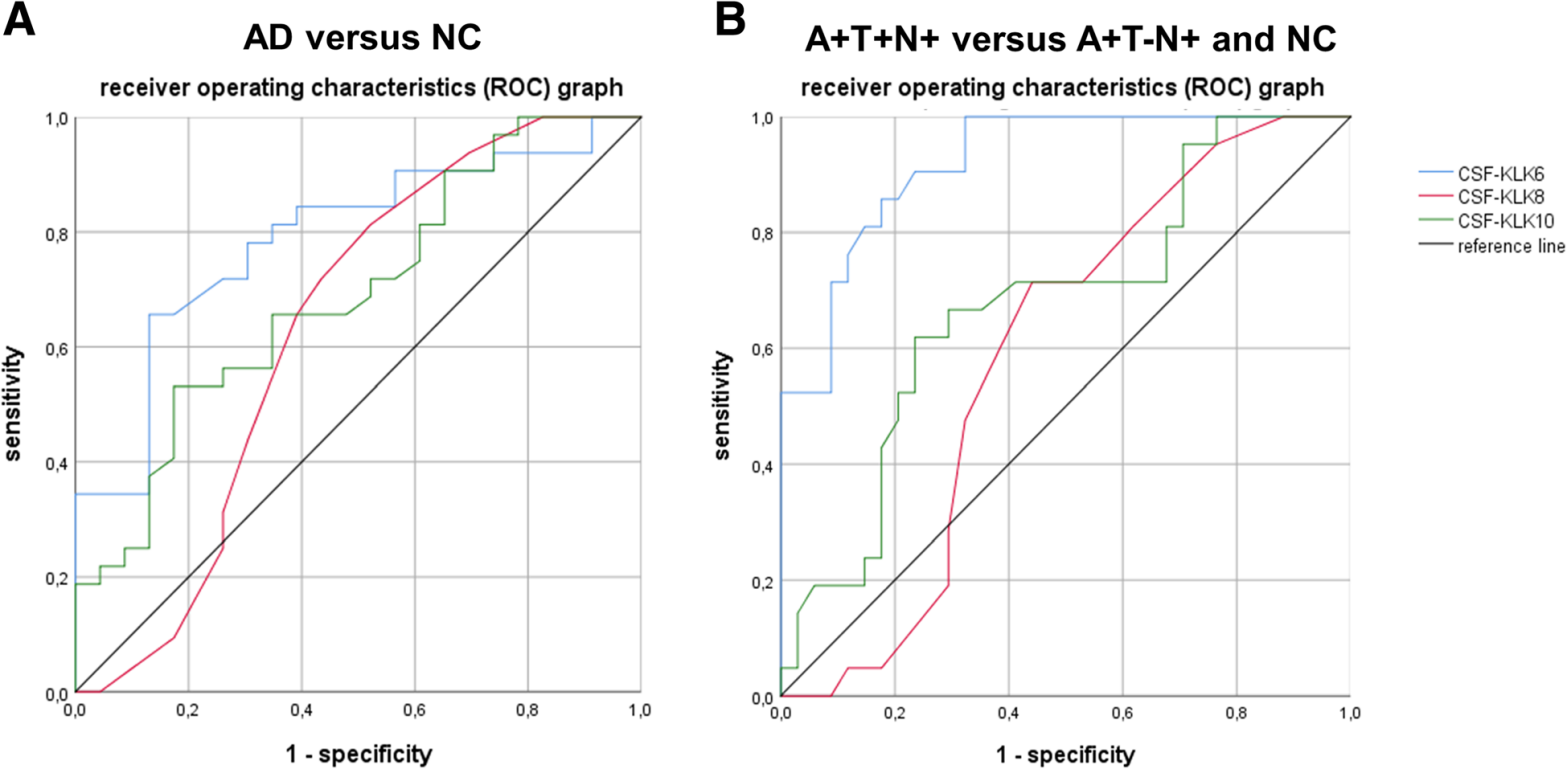
ROC curves of KLK6, KLK8 and KLK10 in CSF between patients with AD and NCs. **a** ROC graph with AUC of KLK6, KLK8 and KLK10 in the CSF between patients with AD and NCs. AUCs were 0.788 (95%-confidence interval (CI) 0.667–0.910) for KLK6, 0.634 (95%-CI 0.471–0.797) for KLK8, and 0.692 (95%-CI 0.552–0.833) for KLK10, respectively. The best cut-off of 270 ng/l for KLK6 had a sensitivity of 66% and a specificity of 87% (LR_AD_ = 7.58, LR_NC_ = 0.39). For KLK8 the best cut-off was 0.145 ng/l (sensitivity 72%, specificity 57%, LR_AD_ = 1.67, LR_NC_ = 0.49) and for KLK10 0.575 ng/l (sensitivity 66%, specificity 65%, LR_AD_ = 1.89, LR_NC_ = 0.52). **b** ROC graph with AUC of KLK6, KLK8 and KLK10 in the CSF between patients with AD (A+/T+/N+) and patients with AD (A+/T−/N+) combined with NC. AUCs were 0.922 (95%-CI 0.855–0.988) for KLK6, 0.591 (95%-CI 0.441–0.741) for KLK8, and 0.679 (95%-CI 0.533–0.826) for KLK10, respectively. The best cut-off was 270 ng/l for KLK6 (sensitivity 86%, specificity 83%, LR_AD A + T + N+_ = 5.06, LR_NC&AD + A + T-N+_ = 0.17). The best cut-off for KLK8 was 0.155 ng/L (sensitivity 71%, specificity 56%, LR_AD A + T + N+_ = 1.61, LR_NC&AD + A + T-N+_ = 0.52) and for KLK10 (sensitivity 62%, specificity 77%, LR_AD A + T + N+_ = 2.70, LR_NC&AD + A + T-N+_ = 0.49). ROC: receiver operating characteristics, AUC: area under the curve

ROC curves for discrimination between AD and NC were calculated (Fig. 2a) with AUCs of 0.788 for KLK6, 0.634 for KLK8 and 0.692 for KLK10, respectively. In the ANOVA model controlled for age, sex, and ApoE ɛ4 allele frequency, (and interaction term AD_y/n_ x ApoEε4 frequency in the KLK10 model), the factor AD_y/n_ was significant using KLK6 or KLK10, but not KLK8, as dependent variable: KLK6 model: adjust. *R*^*2*^ = 0.232, factor AD_y/n_: *p* = 0.025; KLK10 model: adjust. *R*^*2*^ = 0.107, factor AD_y/n_: *p* = 0.042; KLK8 model: adjust. *R*^*2*^ = − 0.033, factor AD_y/n_: *p* = 0.424. Hence, the differences of KLK6 and KLK10 between groups observed in the t-tests were confirmed in the ANOVA analyses controlling for confounders. Additionally, in a posthoc age-matched cohort of AD (*n* = 15) an NC (*n* = 15) KLK6 and KLK10 were significantly different among both (KLK6: *p* < 0.012, KLK8: *p* = 0.250, KLK10: *p* = 0.017).

KLK6, KLK8 and KLK10 were not significantly associated with clinical severity measured through MMSE and global CDR in AD (see Additional file 1: Table S2).

### A/T/(N) system

**Fig. 3:**
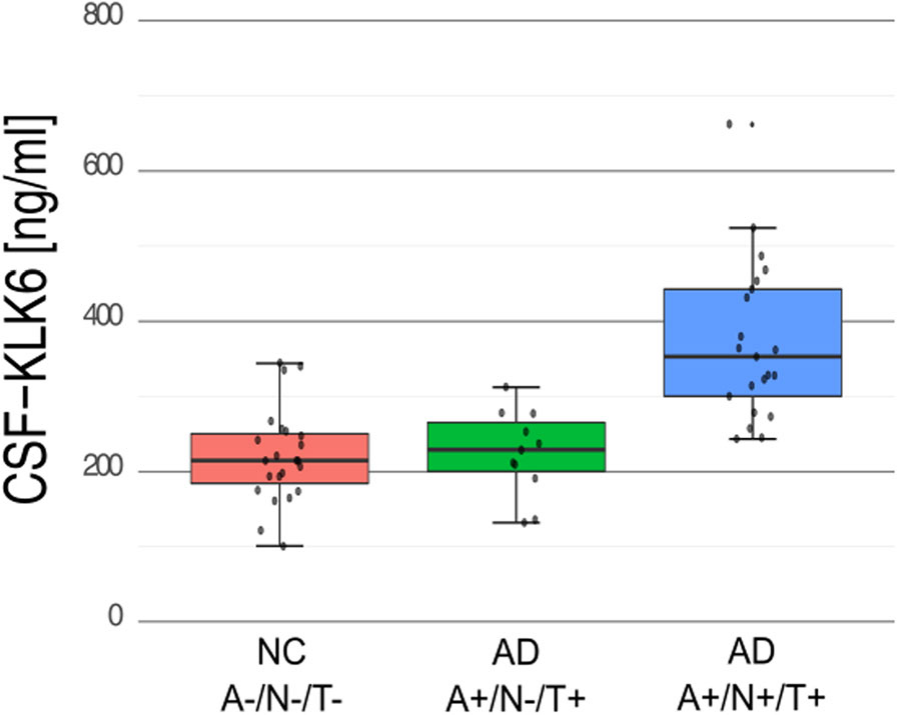
Levels of CSF-KLK6 in CSF of NC and patients with AD stratified to the A/T/(N) system. Levels of CSF-KLK6 in CSF of NC and patients with AD stratified to the A/T/(N) system. KLK6 levels significantly differed between AD A+/T+/N+ and AD A+/T−/N+ (*p* = 0.001) or NC A−/T−/N- (*p* < 0.001), respectively, but not between AD A+/T−/N+ and AD A−/T−/N- (*p* = 0.768). AD: patients with Alzheimer’s disease, NC: cognitively normal controls, A: amyloid pathology, T: tau pathology, N: neuronal injury, A/T/(N) system [54]

The sample was stratified into the AD groups A+/T+/N+ (*n* = 21) and A+/T−/N+ (*n* = 11) and into the NC group A−/T−/N- (*n* = 23) (see Table 2). All KLKs were normally distributed in all subgroups (data not shown). In t-test comparison between NC A-T-N- and AD A + T + N+, KLK6 (*p* < 0.001) and KLK10 (*p* = 0.005) differed significantly but not KLK8 (*p* = 0.683), whereas KLKs between NC A-T-N- and AD A + T-N+ did not (p_KLK6_ = 0.768, p_KLK8_ = 0.649 and p_KLK10_ = 0.156). Between AD A + T-N+ and AD A + T + N+, KLK6 levels were significantly different (*p* = 0.001), but neither KLK8 (*p* = 0.829) nor KLK10 (*p* = 0.366). Box plots for CSF-KLK6 are shown at Fig. 3. In ROC statistic, CSF-KLK6 showed a very high AUC of 0.992 distinguishing A+/T+/N+ from NC A−/T−/N- and AD A+/T−/N+ (Fig. 2b). An additional stratification based on biomarker concordance, i.e. amyloid positivity on both PET and CSF, and neurodegeneration positivity on atrophy in cMRI, CSF-tTau and AD-like hypometabolism in FDG-PET is depicted in Table 3.

**Table 2:** Levels of KLK6, KLK8 and KLK10 in CSF of NC and patients with AD stratified to the A/T/(N) system

	AD A+/T+/N+ (*n* = 21)	AD A+/T−/N+ (*n* = 11)	NC A−/T−/N- (*n* = 23)
CSF-KLK6 ng/ml	372 ± 106.2 (243–663)	224 ± 56.7 (132–312)	220 ± 62.4 (101–344)
CSF-KLK8 ng/ml	0.16 ± 0.024 (0.12–0.21)	0.16 ± 0.031 (0.12–0.22)	0.16 ± 0.058 (0.10–0.37)
CSF-KLK10 ng/ml	0.69 ± 0.220 (0.32–1.11)	0.61 ± 0.26 (0.22–1.03)	0.48 ± 0.249 (0.08–0.95)

*KLK* Kallikrein-related peptidase, *AD* Alzheimer’s dementia, *NC* normal controls, *A* amyloid pathology, *T* Tau pathology, *N* Neuronal injury

**Table 3:** Patients with dementia due to AD stratified according to the A/T/N

A/T/(N) system	Biomarker group	AD biomarker concordance	n
A+/T+/N+ (*n* = 21)	A+	Concordance between amyloid PET and CSF-Aβ_42_	10
		Either amyloid PET or CSF-Aβ_42_ positivity	3
		Only CSF-Aβ_42_ available	8
	N+	Concordance between FDG PET, CSF-tTau and atrophy (cMRI)	18
		Concordance between atrophy (cMRI) and either FDG-PET or CSF-tTau positivity	3
A+/T−/N+ (*n* = 11)	A+	Concordance between amyloid PET and CSF-Aβ_42_	5
		Either amyloid PET or CSF-Aβ_42_ positivity	2
		Only CSF-Aβ_42_ available	4
	N+	Concordance between FDG PET, CSF-tTau and atrophy (cMRI)	6
		Concordance between atrophy (cMRI) and either FDG-PET or CSF-tTau positivity	3

*A+* Amyloid pathology, *T* Tau pathology, *N* Neuronal injury, *PET* Position emission tomography, *CSF* Cerebral spinal fluid, *cMRI* cranial magnetic resonance imaging, *FDG-PET* [18F]fluorodeoxyglucose-position emission tomography, *Aβ* amyloid β

### Associations of KLKs with AD biomarkers

a) In AD: The univariate linear regression analysis with the dependent variable CSF-tTau and KLK6 as the independent variable was significant (adjust. *R*^*2*^ = 0.366, β _KLK6_ = 0.622, *p* < 0.001). Comparable results were received using CSF-pTau levels as dependent variable (adjust. *R*^*2*^ = 0.440, β _KLK6_ = 0.680, *p* < 0.001). All other univariate or multivariate analyses using t-Tau, p-Tau or Aβ_42_, respectively, with KLK-6, KLK-8 or KLK-10, respectively, did neither attain any statistical significance nor increased prediction strength of the models.

b) In NC: The univariate regression analysis with the dependent variable CSF-Aβ_42_ and the independent variable KLK8 was statistically significant (adjust. *R*^*2*^ = 0.192; β_KLK8_ = − 0.478, *p* = 0.021). The best multivariate model additionally included the covariates age, sex and ApoE ɛ4 allele frequency (adjusted *R*^*2*^ = 0.427, p_model_ = 0.006; β_KLK8_ = − 0.586 (*p* = 0.002), β_sex_ = − 0.403 (*p* = 0.026), β_age_ = 0.272 (*p* = 0.109) and β_ApoE_ = − 0.269 (*p* = 0.114)). The model thus explains 42.7% of the variability of CSF-Aβ_42_ levels and showed a significant negative association of KLK8 with CSF-Aβ_42_ levels. The other univariate analyses using t-Tau, p-Tau or Aβ_42_, respectively, with KLK-6, KLK-8 or KLK-10, respectively, did not attain statistical significance.

Correlation analyses between CSF-tTau, CSF-p-Tau and CSF-Aβ42 with each KLK can be overseen in Additional file 1: Table S1.

### Exploratory analyses

Linear regression models with the global FDG-PET or PiB-PET signal to reference region (cerebrum (C) to cerebellum or to pons or to thalamus ratios) as dependent variables with KLK6, KLK8 or KLK10 as independent variables, respectively, were calculated in a sub-cohort of 14 AD patients. Global C/vermis-[^11^C]PiB-PET signal, and C/cerebellum-, C/thalamus and C/pons [^18^F]FDG-PET signal were all normally distributed (Kolmogorow-Smirnow test *p* = 0.148, *p* = 0.200, *p* = 0.200 and *p* = 0.200, respectively). The associations were not significant (Additional file 1: Table S3).

**Fig. 4:**
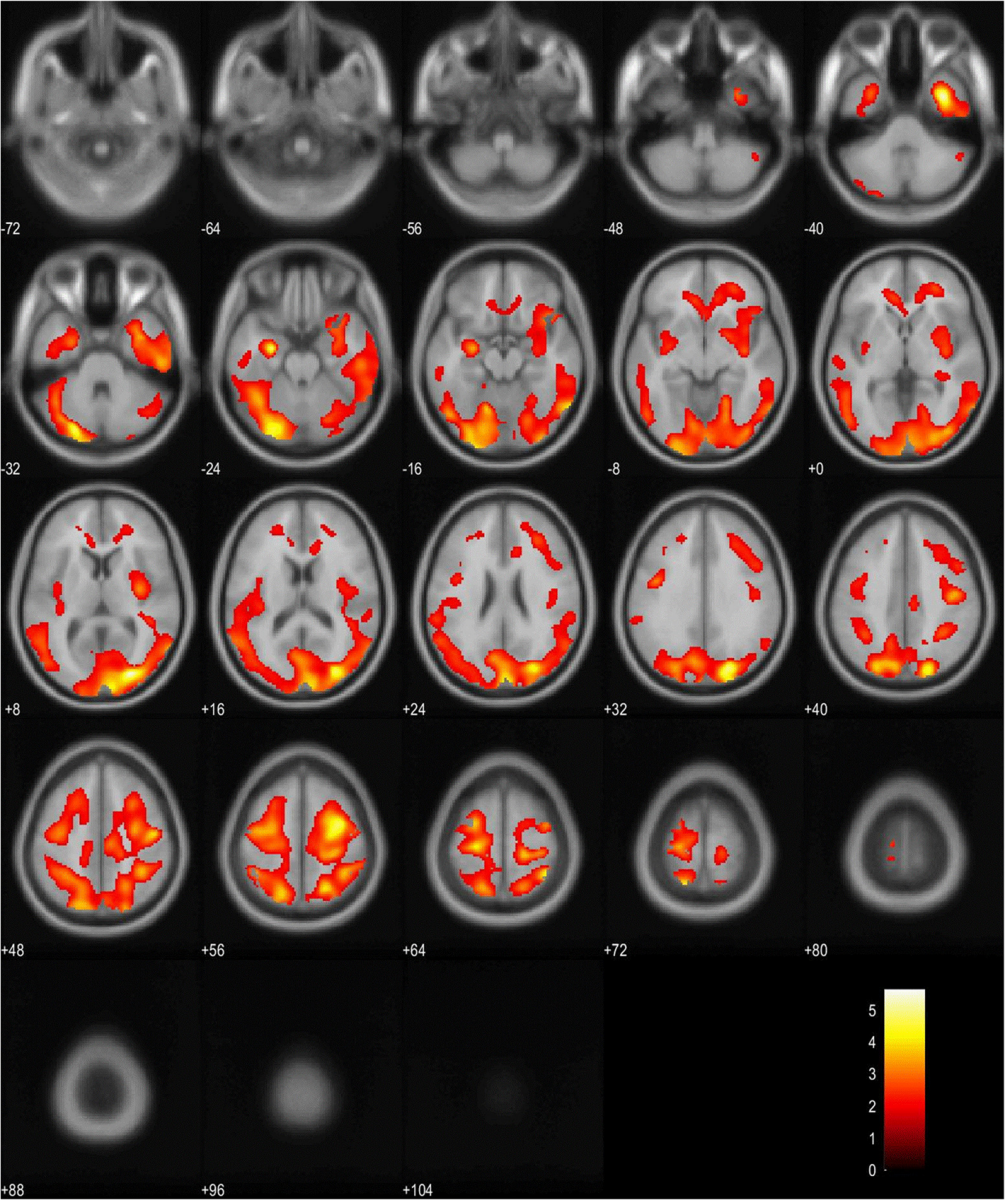
Regional regression analysis between CSF-KLK6 and the regional C/vPiB-PET uptake signal. Voxel based regional regression analysis between CSF-KLK6 levels and the cerebral C/vPiB-PET signal, controlled for copies of the ApoE ɛ4 allele. Significant (*p* < 0.05 FDR corrected) negative correlations are depicted in red to yellow (increasing T values) and are projected on axial T1 MRI scans (average of 152 scans, implemented in SPM12), numbers indicate z-coordinates of slices in Talairach space in mm. CSF: cerebrospinal fluid; KLK: kallikrein-related peptidase, ApoE ɛ4: apolipoprotein E epsilon 4; C/vPiB-PET: Cerebrum to vermis Pittsburgh Compound B-positron emission tomography

The regional voxel-based regression analysis with the regional C/vPiB-PET uptake ratio as the dependent variable and KLK6 as independent variable was not significant. Inclusion of ApoE ɛ4 allele frequency as independent variable into the model resulted in a significant negative association between the CSF-KLK6 levels and the regional amyloid PET signal in the occipital, parietal and temporal cortical areas of both sides (Fig. 4). The regional voxel-based regression analyses with the regional C/vPiB-PET uptake ratio as the dependent variable and KLK8 or KLK10 as independent variables were not significant, even if covariates were included into the models.

**Fig. 5:**
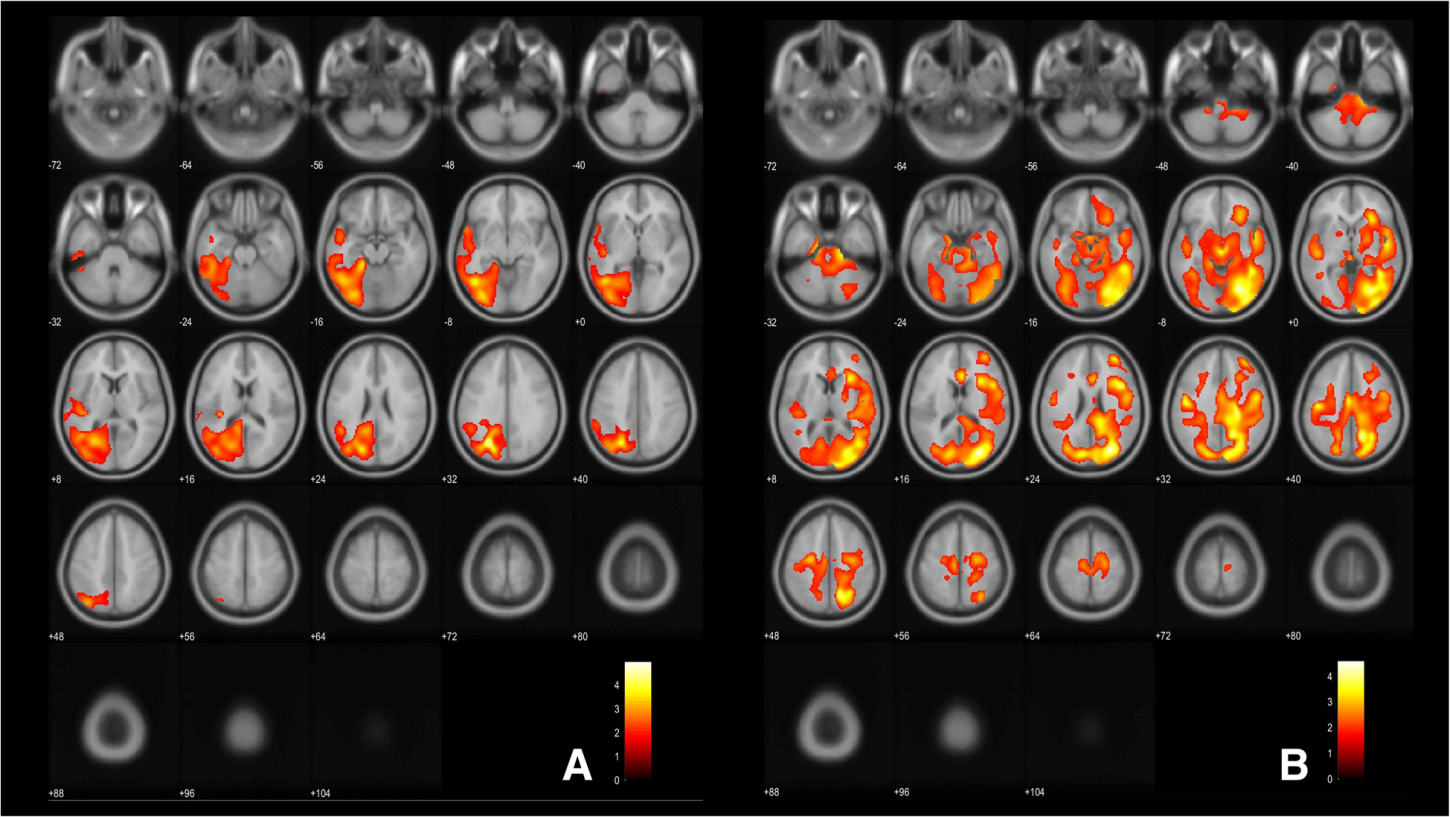
Regional regression analyses between CSF-KLK6 and CSF-KLK10, respectively, and the regional FDG-PET uptake signal. Voxel based regional regression analysis between CSF-KLK6 (**a**) and KLK10 (**b**) and the FGD-PET signal. **a** Significant (*p* < 0.05 FDR corrected) positive associations of KLK6 with the C/p [^18^F]FDG-PET signal and **b** KLK10 with the C/th [^18^F]FDG-PET signal. Correlations are depicted in red to yellow (increasing T values) and are projected on axial T1 MRI scans (average of 152 scans, implemented in SPM12), numbers indicate z-coordinates of slices in Talairach space in mm. For exact cluster distribution compare Additional file 1: Table 4B-C. CSF: cerebrospinal fluid; KLK: kallikrein-related peptidase, ApoE: apolipoprotein E; C/p: Cerebrum to pons; C/th: Cerebrum to thalamus; [^18^F]FDG-PET: [^18^F]fluorodeoxyglucose-position emission tomography

The CSF-KLK6 levels were significantly and positively associated with the C/p [18F]FDG-PET signal predominantly in the left temporal, parietal and occipital cortical areas (Fig. 5a). For a regional overlay of the associations of the CSF-KLK6 levels with the FDG and amyloid PET signal compare Additional file 1: Figure S1, and for exact cluster distribution compare Additional file 1: Table 4A. The CSF-KLK6 levels were neither significantly positively associated with the C/c [18F]FDG-PET nor with the C/th [18F]FDG-PET signal. No significant inverse associations were found between CSF-KLK6 levels and the C/c [18F]FDG-PET, the C/th [^18^F]FDG-PET, or C/p [^18^F]FDG-PET signal, respectively. Also, the CSF-KLK10 levels were significantly and positively associated with the C/th [18F]FDG-PET signal, predominantly in the right in the occipital, temporal and lingual cortex (Fig. 5b). The CSF-KLK8 levels were not significantly associated with the C/c [^18^F]FDG-PET, the C/p [^18^F]FDG-PET or the C/th [^18^F]FDG-PET signal, respectively.

The results of the exploratory analyses on correlations of KLKs with each other and with BBB integrity (including Ralb (ratio of CSF to serum albumin), immunoglobulin G CSF to serum ratios or glucose in the CSF) are provided in the Additional file 1.

## Discussion

KLK6, KLK8 and KLK10 were measured in CSF of patients with clinical diagnosis of AD and evidence of the pathophysiological process, as assessed by a CSF biomarker profile and of a group of subjectively and objectively cognitively normal control subjects with normal AD CSF biomarkers.

### KLK6 and diagnostic potential for prodromal AD

In our study, mean values for CSF-KLK6 levels were significantly different between diagnostic groups (NC vs. AD). After controlling for age, sex, and ApoE ε4 frequency, the group differences remained significant. The obtained AUC for KLK6 was 0.788 which represents a value close to > 0.8 that has been discussed as desired value for an optimal AD biomarker [55]. significant effect of age on KLK group differences was excluded.

In a cross-sectional study, Patra and colleagues [24] did neither find group differences of CSF-KLK6 between subjective cognitive impairment (*n* = 43) and AD (*n* = 43), nor between clinically diagnosed normal controls (*n* = 58) and clinically diagnosed progressive MCI *n* = 28). However, their groups were imbalanced for age, NC groups probably included patients with asymptomatic AD, as indicated by decreased levels of CSF-Aβ and elevated levels of CSF-tTau and CSF-pTau, and MCI and AD patients were not selected for a AD typical biomarker profile. These factors were thoroughly controlled for in our study which may explain the different results. As CSF-KLK6 was increased in early AD, as indicated in the present study, it might be useful for early diagnosis of AD.

### KLK6 and Alzheimer’s pathology

KLK6 is thought to be a predominantly neuron derived protease. After cell death indicated through CSF tau levels, KLK6 might be diluted into CSF like many other proteins [56] and therefore be associated with neuronal injury. Underlining its brain-derived origin, of all measured KLKs only CSF-KLK6 was not significantly associated with the markers of BBB integrity R_alb_s and IgG ratios in AD and NC. Consistently, in AD patients, KLK6 was positively associated with markers of Tau pathology (CSF-pTau) and neuronal injury (CSF-tTau), and after correction for multiple testing, illustrating its similar potential as a marker for neurodegeneration. However, it cannot be ruled out, that the association of CSF-KLK6 with the CSF-pTau protein concentrations is pointing to an involvement in AD pathophysiology, as the following discussion suggests: CSF-KLK6 levels are significantly increased in patients with AD with amyloid positivity and elevated CSF-pTau (A+/T+/N+), the latter a marker specific for neurofibrillary tangles [57], but not in patients with AD with amyloid positivity and normal CSF-pTau levels (A+/T−/N+) or NC (A−/T−/N-). Moreover, KLK-6 discriminated AD A+/T+/N+ from the rest of the cohort with very high accuracy. The association of CSF KLK6 with CSF pTau indicates a specific involvement of KLK6 in AD pathophysiology.

Also, in the subgroup of patients with FDG-PET data, CSF-KLK6 levels were significantly associated with the regional FDG-PET uptake ratio predominantly in the occipital and left temporal areas. To interpretate this association, the assumption that CSF-KLK6 levels correlate with the KLK6 in the brain can be made [56]. Considering that FDG metabolism in the occipital lobe is relatively preserved in AD, it is tempting to speculate that increased levels of CSF-KLK6 could contribute to this observation. This hypothesis is strengthened by the fact that KLK6 levels were inversely associated with amyloid load in this area indicating a beneficial effect of KLK6 on toxic amyloid accumulation. KLK6 is also expressed in endothelial cells at the choroid plexus [12], at intracerebral vessels [15], and at the BBB itself [12]. Its location at the neurovascular unit and close to the amyloid clearing perivascular spaces makes KLK6 a suspect to be involved in the amyloid clearance process or immunological processes that impair amyloid clearance via perivascular drainage [58]. The inverse association of CSF-KLK6 with cerebral amyloid in the occipital lobes has first to be confirmed in further samples. However, also another anti-amyloidgenic protease like KLK6, the transmembrane protease Neprilysin, is higher expressed in the occipital lobe with relatively dense distribution in the primary somatosensory and visual cortices compared with the hippocampus and association cortices [59]. In AD, Neprilysin expression is inversely associated with amyloid levels in regions vulnerable to senile plaque development, such as hippocampus and midtemporal gyrus [60]. Somehow, if KLK6 is, indeed, anti-amyloidgenic, it seems that the occipital lobe is relatively protected by an arsenal of enzymes against amyloid.

There is in vitro evidence that KLK6 cleaves the amyloid precursor protein (APP) at three sites: firstly, one amino acid before the β-secretase site (BACE), secondly, five amino acids after the N-terminal end of Aβ peptide sequence and thirdly, identical to the α-secretase site [61]. This data is based on artificial APP-like monomers in-vitro. KLK6 might reduce Aβ monomers through its α-secretase-like function [20]. KLK6 might also cause truncated (N-terminal) Aβ peptides that are discussed to facilitate plaque formation [61]. This is in line with the finding of Little at al. showing amyloidogenic fragments in cells co-transfected with KLK6 and APP [20]. In summary, the association between KLK6 and APP products is still under debate and needs to be elucidated in future studies. KLK8 and KLK 10 are not known to cleave any of the AD biomarkers.

### KLK8 and Alzheimer’s pathology

CSF-KLK8 levels are negatively associated with CSF-Aβ_42_ levels in NC, but not in AD. In accordance with our finding, a positive association between KLK8 levels and amyloid load was detected in the hippocampus only in early disease stages of AD mice, when inhibition of KLK8 led to increased amyloid clearance, but not in the further course of the disease [30]. This points to an amyloid modulating property of KLK8 limited to healthy or early disease states. In human AD, other processes influencing amyloid clearance or KLK levels might attenuate the association between KLK8 and Aβ_42_ levels. Whereas levels of KLK8 are higher in female AD mice before amyloid accumulation begins and are potentially also higher in female human brains [31] we could not find any effect of sex concerning KLK8 levels in CSF.

### KLK10 and Alzheimer’s pathology

In the case of KLK10, mean values were also significantly different between groups, but with a smaller AUC (0.692) as compared to KLK6. So far, there is no data linking KLK10, in particular CSF levels, pathologically to AD. Here, we show that KLK10 levels are significantly increased in AD, and that they are positively associated with the regional glucose metabolism in the temporal and occipital areas comparable to the associations observed with KLK6. Further research is needed to elucidate the role of KLK10 in AD. In line with Diamandis et al. [33] reporting higher CSF-KLK10 levels in AD compared to clinically diagnosed normal controls, we now confirm these results in a psychometricly tested and biomarker-based cohort. It is interesting that patients with FTD show significantly lower levels of KLK10 than patients with AD [33]. Thus, there might be an additional clinical benefit in assessing CSF-KLK10 to distinguish between FTD and AD.

### KLKs involved in matrix degradation and inflammatory processes

In contrast to CSF-KLK6, CSF-KLK8 and CSF-KLK10 are significantly associated with the markers of BBB integrity R_alb_s and IgG ratios in AD and NC in our study, pointing to a potential secondary non-brain origin [62]. KLK8 and potentially KLK10 are cleaving several proteins of the extracellular matrix (ECM) in the nervous system like fibronectin, casein, fibrinogen and collagen IV [21, 63, 64] and might lead to BBB disturbances, i. e. at perivascular spaces. Whilst in AD impaired drainage alongside perivascular spaces is a discussed cause of AD [65], it is not of FTD. This might explain lower levels of CSF-KLK10 in FTD compared to AD. CSF-KLK8 and CSF-KLK10 were not significantly associated with tau or amyloid pathology in AD. Therefore, leakage of these proteins from dying cells or a direct involvement in AD CSF biomarkers as a cause for elevated CSF KLK levels is unlikely.

KLK6 can also cleave ECM proteins, but the functional specificity among KLKs differs significantly. It is interesting, that KLK6, expressed by oligodendrocytes, indirectly influences the permeability of the BBB and increases inflammatory responses [66]. This might be induced by a KLK6 mediated activation of the proteinase-activated receptors (PARs) that are involved in inflammation [67]. CSF-KLK8 and CSF-KLK10 might also be linked to this immune reaction, since they co-locate in immune-associated tissues [8]. Furthermore, KLK6 also cleaves myelin basic protein and is involved in myelin turnover [68]. Damage to oligodendrocytes may cause elevated CSF-KLK6, as in inflammatory diseases like MS [69]. As an inflammatory component is also included in AD, elevation of KLK6 might represented a biomarker for inflammatory reactions in the brain.

### Patient’s cohort and limitation

We did not find any significant associations of KLKs with clinical severity (MMSE and global CDR). However, our AD group suffered from early AD. Hence, our results might not be generalizable to advanced stages of dementia. When we excluded the one NC with a pathological CSF tTau level, there was no perceptible difference (post hoc) in analyses (data not shown).

## Conclusions

KLK6, KLK8 and KLK10 are likely to play a general role in neurodegenerative processes in the CNS including the disruption of the BBB, cell death, and inflammatory processes, and potentially in amyloid pathology itself. Especially CSF-KLK6 is associated with Tau pathology, as indicated by elevated CSF p-tau levels in AD, and has the potential to be a suitable biomarker for detecting AD patients with tau pathology, for diagnosis, monitoring and/ or prognosis. In addition, KLK6 is associated with regional cerebral amyloid load and glucose metabolism. Future studies need to address its specificity in distinguishing between other neurodegenerative diseases.

## Additional file

Additional file 1: Supplementary PET methods (1. Brain imaging),  analyses (2. Association of KLKs with BBB integrity), results (3. Associations of KLKs with the global PET signal, correlations of KLKs with each other and associations of KLKs with BBB integrity), tables (4.), figures (5.) and references (6.). **Table S1:** Correlations of KLK6, KLK8 and KLK10 with AD biomarkers in CSF in AD and NC; **Table S2:** Associations of KLKs and clinical severity; **Table S3:** Univariate linear regression models with associations of PET-AD-biomarkers with KLK6, KLK8 and KLK10 in a sub cohort of 14 AD patients; **Table S4:** A) Cluster distribution of the association of CSF-KLK6 with the cerebral amyloid controlled for ApoE ε4 allele frequency; B) Cluster distribution of the association of CSF-KLK6 with the cerebral glucose metabolism; C) Cluster distribution of the association of CSF-KLK10 with the cerebral glucose metabolism. **Figure S1:** Overlay of significant clusters of the associations of CSF-KLK6 with the FDG-PET and PiB-PET signal. (DOCX 346 kb)

## Data Availability

The datasets used and analyzed during the present study are available from the corresponding author on reasonable request.

## Abbreviations

[^11^C]PiB: Pittsburgh Compound B
[^18^F]FDG-PET: [^18^F]Fluoro-D-glucose PET
AD: Alzheimer’s disease
ApoEε4: Apolipoprotein E epsilon 4 allele frequency
APP: Amyloid precursor protein
Aβ_42_: Amyloid 1–42
BACE: β-secretase
BBB: Blood-brain barrier
C: Cerebrum
CAA: Cerebral amyloid angiopathy
CDR: Dementia rating scale
CNS: Central nervous system
CSF: Cerebrospinal fluid
CVs: Coefficients of correlation
ECM: Extracellular matrix
ELISA: Enzyme-linked immunosorbent assays
EPHB2: Epinephrine receptor B2
FLAIR: Fluid-Attenuated inverse recovery
FTD: Frontotemporal dementia
hAPP: Human amyloid precursor protein
KLK: Kallikrein-like peptidase
LRP1: Lipoprotein-receptor related protein 1
MMSE: Mini-Mental State Examination
MOCA: Montreal Cognitive Assessment
MRI: Magnetic resonance imaging
MS: Multiple sclerosis
NC: Cognitive normal control
PAR: Proteinase-activated receptor
PET: Positron emission tomography
pTau: Phosphor Tau
ROC: Receiver-operating characteristics
SD: Standard deviation
SOB: Sum of boxes
tTau: Total Tau

## References

[1] Tarasoff-Conway JM, Carare RO, Osorio RS, Glodzik L, Butler T, Fieremans E (2015). Clearance systems in the brain-implications for Alzheimer disease. Nat Rev Neurol.

[2] Thal DR, Rüb U, Orantes M, Braak H (2002). Phases of a beta-deposition in the human brain and its relevance for the development of AD. Neurology.

[3] Hoffman JM, Welsh-Bohmer KA, Hanson M, Crain B, Hulette C, Earl N (2000). FDG PET imaging in patients with pathologically verified dementia. J Nucl Med Off Publ Soc Nucl Med.

[4] Tapiola T, Alafuzoff I, Herukka S-K, Parkkinen L, Hartikainen P, Soininen H (2009). Cerebrospinal fluid {beta}-amyloid 42 and tau proteins as biomarkers of Alzheimer-type pathologic changes in the brain. Arch Neurol.

[5] Ossenkoppele R, Jansen WJ, Rabinovici GD, Knol DL, van der Flier WM, van Berckel BNM (2015). Prevalence of amyloid PET positivity in dementia syndromes. JAMA.

[6] Prassas I, Eissa A, Poda G, Diamandis EP (2015). Unleashing the therapeutic potential of human kallikrein-related serine proteases. Nat Rev Drug Discov.

[7] Pericak-Vance MA, Bebout JL, Gaskell PC, Yamaoka LH, Hung W-Y, Alberts MJ (1991). Linkage studies in familial Alzheimer disease: evidence for chromosome 19 linkage. Am J Hum Genet.

[8] Shaw JLV, Diamandis EP (2007). Distribution of 15 human Kallikreins in tissues and biological fluids. Clin Chem.

[9] Shimizu-Okabe C, Yousef GM, Diamandis EP, Yoshida S, Shiosaka S, Fahnestock M (2001). Expression of the kallikrein gene family in normal and Alzheimer’s disease brain. Neuroreport.

[10] Kalinska M, Meyer-Hoffert U, Kantyka T, Potempa J (2016). Kallikreins - the melting pot of activity and function. Biochimie.

[11] Diamandis EP, Yousef GM, Soosaipillai AR, Grass L, Porter A, Little S (2000). Immunofluorometric assay of human kallikrein 6 (zyme/protease M/neurosin) and preliminary clinical applications. Clin Biochem.

[12] Diamandis EP, Yousef GM, Petraki C, Soosaipillai AR (2000). Human Kallikrein 6 as a biomarker of Alzheimer’s disease. Clin Biochem.

[13] Mitsui S, Okui A, Uemura H, Mizuno T, Yamada T, Yamamura Y (2002). Decreased cerebrospinal fluid levels of Neurosin (KLK6), an aging-related protease, as a possible new risk factor for Alzheimer’s disease. Ann N Y Acad Sci.

[14] Petraki CD, Karavana VN, Skoufogiannis PT, Little SP, Howarth DJ, Yousef GM (2001). The spectrum of human kallikrein 6 (zyme/protease M/neurosin) expression in human tissues as assessed by immunohistochemistry. J Histochem Cytochem Off J Histochem Soc.

[15] Ashby EL, Kehoe PG, Love S (2010). Kallikrein-related peptidase 6 in Alzheimer’s disease and vascular dementia. Brain Res.

[16] Burda JE, Radulovic M, Yoon H, Scarisbrick IA (2013). Critical role for PAR1 in Kallikrein 6-mediated Oligodendrogliopathy. Glia.

[17] Scarisbrick IA, Isackson PJ, Ciric B, Windebank AJ, Rodriguez M (2001). MSP, a trypsin-like serine protease, is abundantly expressed in the human nervous system. J Comp Neurol.

[18] Scarisbrick IA, Radulovic M, Burda JE, Larson N, Blaber SI, Giannini C (2012). Kallikrein 6 is a novel molecular trigger of reactive astrogliosis. Biol Chem.

[19] Iwata A, Maruyama M, Akagi T, Hashikawa T, Kanazawa I, Tsuji S (2003). Alpha-synuclein degradation by serine protease neurosin: implication for pathogenesis of synucleinopathies. Hum Mol Genet.

[20] Little SP, Dixon EP, Norris F, Buckley W, Becker GW, Johnson M (1997). Zyme, a novel and potentially amyloidogenic enzyme cDNA isolated from Alzheimer’s disease brain. J Biol Chem.

[21] Shimizu C, Yoshida S, Shibata M, Kato K, Momota Y, Matsumoto K (1998). Characterization of recombinant and brain Neuropsin, a plasticity-related serine protease. J Biol Chem.

[22] Zarghooni M, Soosaipillai A, Grass L, Scorilas A, Mirazimi N, Diamandis EP (2002). Decreased concentration of human kallikrein 6 in brain extracts of Alzheimer’s disease patients. Clin Biochem.

[23] Dukic L, Simundic A-M, Martinic-Popovic I, Kackov S, Diamandis A, Begcevic I (2016). The role of human kallikrein 6, clusterin and adiponectin as potential blood biomarkers of dementia. Clin Biochem.

[24] Patra K, Soosaipillai A, Sando SB, Lauridsen C, Berge G, Møller I, et al. Assessment of kallikrein 6 as a cross-sectional and longitudinal biomarker for Alzheimer’s disease. Alzheimers Res Ther. 2018;10(1):9. 10.1186/s13195-018-0336-4 PMC5789599 29378650

[25] Attwood B, Bourgognon J-M, Patel S, Mucha M, Schiavon E, Skrzypiec AE (2011). Neuropsin cleaves EphB2 in the amygdala to control anxiety. Nature.

[26] Cissé M, Halabisky B, Harris J, Devidze N, Dubal DB, Sun B (2011). Reversing EphB2 depletion rescues cognitive functions in Alzheimer model. Nature.

[27] Jiang J, Wang Z-H, Qu M, Gao D, Liu X-P, Zhu L-Q, et al. Stimulation of EphB2 attenuates tau phosphorylation through PI3K/Akt-mediated inactivation of glycogen synthase kinase-3β. Sci Rep. 2015;5 Available from: http://www.ncbi.nlm.nih.gov/pmc/articles/PMC4484244/. [cited 2017 Feb 23]. 10.1038/srep11765 PMC4484244 26119563

[28] Qu M, Jiang J, Liu X-P, Tian Q, Chen L-M, Yin G (2013). Reduction and the intracellular translocation of EphB2 in Tg2576 mice and the effects of β-amyloid. Neuropathol Appl Neurobiol.

[29] Simón AM, de Maturana RL, Ricobaraza A, Escribano L, Schiapparelli L, Cuadrado-Tejedor M (2009). Early changes in hippocampal Eph receptors precede the onset of memory decline in mouse models of Alzheimer’s disease. J Alzheimers Dis JAD.

[30] Herring A, Münster Y, Akkaya T, Moghaddam S, Deinsberger K, Meyer J (2016). Kallikrein-8 inhibition attenuates Alzheimer’s disease pathology in mice. Alzheimers Dement J Alzheimers Assoc.

[31] Keyvani K, Münster Y, Kurapati NK, Rubach S, Schönborn A, Kocakavuk E (2018). Higher levels of kallikrein-8 in female brain may increase the risk for Alzheimer’s disease. Brain Pathol Zurich Switz.

[32] Petraki CD, Karavana VN, Luo L-Y, Diamandis EP (2002). Human kallikrein 10 expression in normal tissues by immunohistochemistry. J Histochem Cytochem Off J Histochem Soc.

[33] Diamandis Eleftherios P, Scorilas Andreas, Kishi Tadaaki, Blennow Kaj, Luo Liu-Ying, Soosaipillai Antoninus, Rademaker Alfred W, Sjogren Magnus (2004). Altered kallikrein 7 and 10 concentrations in cerebrospinal fluid of patients with Alzheimer's disease and frontotemporal dementia. Clinical Biochemistry.

[34] Sotiropoulou G, Pampalakis G, Diamandis EP (2009). Functional roles of human Kallikrein-related peptidases. J Biol Chem.

[35] Schain M, Kreisl WC (2017). Neuroinflammation in neurodegenerative disorders-a review. Curr Neurol Neurosci Rep.

[36] Folstein MF, Folstein SE, McHugh PR (1975). “Mini-mental state”. A practical method for grading the cognitive state of patients for the clinician. J Psychiatr Res.

[37] Morris JC, Heyman A, Mohs RC, Hughes JP, van Belle G, Fillenbaum G (1989). The consortium to establish a registry for Alzheimer’s disease (CERAD). Part I. clinical and neuropsychological assessment of Alzheimer’s disease. Neurology.

[38] Morris JC (1993). The clinical dementia rating (CDR): current version and scoring rules. Neurology.

[39] Albert MS, DeKosky ST, Dickson D, Dubois B, Feldman HH, Fox NC (2011). The diagnosis of mild cognitive impairment due to Alzheimer’s disease: recommendations from the National Institute on Aging-Alzheimer’s association workgroups on diagnostic guidelines for Alzheimer’s disease. Alzheimers Dement J Alzheimers Assoc.

[40] McKhann GM, Knopman DS, Chertkow H, Hyman BT, Jack CR, Kawas CH (2011). The diagnosis of dementia due to Alzheimer’s disease: recommendations from the National Institute on Aging-Alzheimer’s association workgroups on diagnostic guidelines for Alzheimer’s disease. Alzheimers Dement J Alzheimers Assoc.

[41] Hulstaert F, Blennow K, Ivanoiu A, Schoonderwaldt HC, Riemenschneider M, De Deyn PP (1999). Improved discrimination of AD patients using beta-amyloid (1-42) and tau levels in CSF. Neurology.

[42] Berres M, Monsch AU, Bernasconi F, Thalmann B, Stähelin HB (2000). Normal ranges of neuropsychological tests for the diagnosis of Alzheimer’s disease. Stud Health Technol Inform.

[43] Zivelin A, Rosenberg N, Peretz H, Amit Y, Kornbrot N, Seligsohn U (1997). Improved method for genotyping apolipoprotein E polymorphisms by a PCR-based assay simultaneously utilizing two distinct restriction enzymes. Clin Chem.

[44] Minoshima S, Frey KA, Koeppe RA, Foster NL, Kuhl DE (1995). A diagnostic approach in Alzheimer’s disease using three-dimensional stereotactic surface projections of fluorine-18-FDG PET. J Nucl Med Off Publ Soc Nucl Med.

[45] Jagust W, Reed B, Mungas D, Ellis W, Decarli C (2007). What does fluorodeoxyglucose PET imaging add to a clinical diagnosis of dementia?. Neurology.

[46] Grimmer T, Henriksen G, Wester HJ, Forstl H, Klunk WE, Mathis CA (2009). Clinical severity of Alzheimer’s disease is associated with PIB uptake in PET. Neurobiol Aging.

[47] Grimmer T, Riemenschneider M, Forstl H, Henriksen G, Klunk WE, Mathis CA (2009). Beta amyloid in Alzheimer’s disease: increased deposition in brain is reflected in reduced concentration in cerebrospinal fluid. Biol Psychiatry.

[48] Grimmer T, Tholen S, Yousefi BH, Alexopoulos P, Forschler A, Forstl H (2010). Progression of cerebral amyloid load is associated with the apolipoprotein E epsilon4 genotype in Alzheimer’s disease. Biol Psychiatry.

[49] Goos JDC, Henneman WJP, Sluimer JD, Vrenken H, Sluimer IC, Barkhof F (2010). Incidence of cerebral microbleeds: a longitudinal study in a memory clinic population. Neurology.

[50] Tsolakidou A, Alexopoulos P, Guo L-H, Grimmer T, Westerteicher C, Kratzer M (2013). β-Site amyloid precursor protein-cleaving enzyme 1 activity is related to cerebrospinal fluid concentrations of sortilin-related receptor with A-type repeats, soluble amyloid precursor protein, and tau. Alzheimers Dement J Alzheimers Assoc.

[51] Luo L-Y, Grass L, Howarth DJC, Thibault P, Ong H, Diamandis EP (2001). Immunofluorometric assay of human Kallikrein 10 and its identification in biological fluids and tissues. Clin Chem.

[52] Kishi T, Grass L, Soosaipillai A, Shimizu-Okabe C, Diamandis EP (2003). Human Kallikrein 8: immunoassay development and identification in tissue extracts and biological fluids. Clin Chem.

[53] Jack CR, Bennett DA, Blennow K, Carrillo MC, Feldman HH, Frisoni GB (2016). A/T/N: an unbiased descriptive classification scheme for Alzheimer disease biomarkers. Neurology.

[54] Jack CR, Bennett DA, Blennow K, Carrillo MC, Dunn B, Haeberlein SB (2018). NIA-AA research framework: toward a biological definition of Alzheimer’s disease. Alzheimers Dement J Alzheimers Assoc.

[55] Khachaturian ZS, Radebaugh TS (1998). Consensus report of the working group on: “molecular and biochemical markers of Alzheimer’s disease” 11The names of the working group members and the names of the working group advisory committee members are listed in the appendix a(section VI). 22The Reagan institute working groups are planned and organized by Z. S. Khachaturian and T.S. Radebaugh; fax: 301-879-2023; E-mail: zaven@idt.net. Neurobiol Aging.

[56] Begcevic I, Brinc D, Drabovich AP, Batruch I, Diamandis EP. Identification of brain-enriched proteins in the cerebrospinal fluid proteome by LC-MS/MS profiling and mining of the Human Protein Atlas. Clin Proteomics. 2016;13 Available from: https://www.ncbi.nlm.nih.gov/pmc/articles/PMC4868024/. 10.1186/s12014-016-9111-3 PMC4868024 27186164

[57] Buerger K, Ewers M, Pirttilä T, Zinkowski R, Alafuzoff I, Teipel SJ (2006). CSF phosphorylated tau protein correlates with neocortical neurofibrillary pathology in Alzheimer’s disease. Brain J Neurol.

[58] Thal DR (2009). The pre-capillary segment of the blood-brain barrier and its relation to perivascular drainage in Alzheimer’s disease and small vessel disease. ScientificWorldJournal.

[59] Akiyama H, Kondo H, Ikeda K, Kato M, McGeer PL (2001). Immunohistochemical localization of neprilysin in the human cerebral cortex: inverse association with vulnerability to amyloid beta-protein (Abeta) deposition. Brain Res.

[60] Yasojima K, McGeer EG, McGeer PL (2001). Relationship between beta amyloid peptide generating molecules and neprilysin in Alzheimer disease and normal brain. Brain Res.

[61] Magklara A, Mellati AA, Wasney GA, Little SP, Sotiropoulou G, Becker GW (2003). Characterization of the enzymatic activity of human kallikrein 6: autoactivation, substrate specificity, and regulation by inhibitors. Biochem Biophys Res Commun.

[62] Keaney J, Campbell M (2015). The dynamic blood-brain barrier. FEBS J.

[63] Rajapakse S, Ogiwara K, Takano N, Moriyama A, Takahashi T (2005). Biochemical characterization of human kallikrein 8 and its possible involvement in the degradation of extracellular matrix proteins. FEBS Lett.

[64] Debela M, Magdolen V, Bode W, Brandstetter H, Goettig P (2016). Structural basis for the Zn2+ inhibition of the zymogen-like kallikrein-related peptidase 10. Biol Chem.

[65] Grimmer T, Faust M, Auer F, Alexopoulos P, Förstl H, Henriksen G (2012). White matter hyperintensities predict amyloid increase in Alzheimer’s disease. Neurobiol Aging.

[66] Bando Y, Hagiwara Y, Suzuki Y, Yoshida K, Aburakawa Y, Kimura T (2018). Kallikrein 6 secreted by oligodendrocytes regulates the progression of experimental autoimmune encephalomyelitis. Glia.

[67] Oikonomopoulou K, Hansen KK, Saifeddine M, Tea I, Blaber M, Blaber SI (2006). Proteinase-activated receptors, targets for Kallikrein signaling. J Biol Chem.

[68] Angelo PF, Lima AR, Alves FM, Blaber SI, Scarisbrick IA, Blaber M (2006). Substrate specificity of human Kallikrein 6 salt and glycosaminoglycan activation effects. J Biol Chem.

[69] Hebb ALO, Bhan V, Wishart AD, Moore CS, Robertson GS (2010). Human kallikrein 6 cerebrospinal levels are elevated in multiple sclerosis. Curr Drug Discov Technol.

