# Supplementary material for: Kallikrein-related peptidases 6 and 10 are elevated in cerebrospinal fluid of patients with Alzheimer’s disease and associated with CSF-TAU and FDG-PET
Source: Transl Neurodegener. 2019 Aug 27;8:25. doi: 10.1186/s40035-019-0168-6 (PMC6712703; doi:10.1186/s40035-019-0168-6)
Supplement: Supplementary file 1 — Supplementary PET methods (1. Brain imaging), analyses (2. Association of KLKs with BBB integrity), results (3. Associations of KLKs with the global PET signal, correlations of KLKs with each other and associations of KLKs with BBB integrity), tables (4.), figures (5.) and references (6.). Table S1: Correlations of KLK6, KLK8 and KLK10 with AD biomarkers in CSF in AD and NC; Table S2: Associations of KLKs and clinical severity; Table S3: Univariate linear regression models with associations of PET-AD-biomarkers with KLK6, KLK8 and KLK10 in a sub cohort of 14 AD patients; Table S4: A) Cluster distribution of the association of CSF-KLK6 with the cerebral amyloid controlled for ApoE ε4 allele frequency; B) Cluster distribution of the association of CSF-KLK6 with the cerebral glucose metabolism; C) Cluster distribution of the association of CSF-KLK10 with the cerebral glucose metabolism. Figure S1: Overlay of significant clusters of the associations of CSF-KLK6 with the FDG-PET and PiB-PET signal. (DOCX 346 kb) [file 40035_2019_168_MOESM1_ESM.docx]

**Additional file**

**1. Supplementary Methods**

Brain imaging (Methods in original article)

PET analyses were performed using standard procedures (4–6). The [^11^C]PiB-PET and [^18^F]FDG-PET images were co-registered to high resolution MRI scans and normalized to the MNI space, using the warping parameters of the MRI to obtain inter-individually comparable images. Using the reference tissue model, relative measures of global cerebral [^11^C]PiB-PET uptake and [^18^F]FDG-PET uptake, respectively, were obtained by calculating a cerebral cortex to cerebellar vermis (C/cv) ratio of each patient’s [^11^C]PiB SUV40-70min scan and cerebral cortex to thalamus (C/t), cortex to pons (C/p) and cerebral to cerebellum (C/c) ratios of each patient's [^18^F]FDG SUV30-50min scan (7) respectively, to control for between-subjects differences in tracer uptake using standard methods (4–6,8). For regional analyses, PET images were normalized to the same reference regions (9).

**2. Supplementary analyses**

Association of KLK2 with BBB integrity (Exploratory analyses in original article)

Additionally, plausibility analyses were performed to investigate whether KLK levels in the CSF are associated with an impaired blood-brain barrier (BBB). To examine, whether KLKs might originate from the CNS or from the periphery, linear regression analyses were calculated with each KLK as dependent variable and R_alb_ (ratio of CSF to serum albumin), immunoglobulin G CSF to serum ratios or glucose in the CSF as independent variables, respectively.

Because KLK8 and KLK10 were associated with R_alb_ as an indicator for BBB integrity of KLKs, R_alb_ was post-hoc included in the previous linear regression models (compare statistical analyses 5): CSF AD biomarker as dependent variables and KLKs as independent variables including all former covariates, and R_alb,_ and an interaction variable R_alb_ x KLK8 or R_alb_ x KLK10, respectively.

**3. Supplementary results**

Associations of KLKs with the global PET signal (Exploratory results in original article)

In univariate analyses, FDG-PET signal or PiB-PET signal, respectively, were not statistically significantly associated with KLK6, KLK8 or KLK10, respectively (see Table S2). In multivariate analyses, only the model with the dependent variable cerebrum to pons FDG-signal ratio and KLK10 as the independent variable was statistically significant, if clinical severity assessed by global CDR (adjusted R²=0.491 (p_model_= 0.010), β_KLK10_= -0.546 (p= 0.025), β_CDR_= -0.733 (p= 0.005)) or MMSE score (adjusted R²= 0.407 (p_model_= 0.023), β_KLK10_= -0.463 (p= 0.060), β_MMST_= 0.658 (p= 0.012)) were also included in the model. In both models, KLK10 was significantly inversely associated with the cerebrum to pons FDG-PET signal.

Correlations of KLKs with each other

In AD, KLK6 and KLK8 (β= 0.218; p= 0.232), KLK6 and KLK10 (β= -0.042; p= 0.819) and KLK8 and KLK10 (β= 0.215; p= 0.238) levels were not significantly associated with each other. In NC, KLK6 and KLK10 (β= 0.436; p= 0.038) as well as KLK8 and KLK10 (β= 0.471; p= 0.023) were significantly and positively associated, but not KLK6 and KLK8 (β= 0.073; p= 0.741). None of the significant associations survived correction for multiple comparisons.

Associations of KLKs with BBB integrity (Exploratory results in original article)

In AD, KLK8 (p= 0.007, β= 0.470) and KLK10 (p= 0.019, β= 0.413) were significantly associated with R_alb_, but not KLK6 (p= 0.917, β= 0.019). The same pattern was observed in NC: KLK6 (p= 0.904, β= 0.037), KLK8 (p= 0.020, β= 0.636) and KLK10 p= 0.029, β= 0.604). The R_alb_ were numerically slightly higher in NC (n= 13, mean= 8.92 x 10^-3^) than in AD (n= 32, mean 6.36 x 10^-3^) but not significantly different (Mann-Whitney-U test: p= 0.139). Four AD patients and zero NC showed values over the upper reference limit of > 11 x 10^-3^ (11), the highest with 16.3 x 10^-3^. No KLK was significantly associated with glucose or lactate in AD or NC after correction for multiple comparisons, but KLK10 was significantly associated with CSF lactate in AD (adjust. R²= 0.319, p< 0.001, β_KLK_10= 0.584).

In AD, KLK8 (p=0.013, β= 0.435) and KLK10 (p= 0.036, β= 0.372) were significantly associated with immunoglobulin G (IgG) CSF to serum ratio (R_IgG_), but not KLK6 (p=0.942, β= 0.031). The same pattern was observed in NC: KLK6 (p= 0.755, β= 0.092), KLK8 (p= 0.033, β= 0.572) and KLK10 (p= 0.029, β= 0.582), respectively. No participant showed an autochthone intracranial IgG synthesis or oligoclonal bands. R_albs_ and R_IgG_ were highly correlated with each other: in NC, a Spearman correlation coefficient of 0.997 (p=< 0.001), and in AD, a correlation coefficient of 0.923 (p< 0.001) were observed.

In NC, when adding the covariates R_alb_ and the interaction variable R_alb_ X KLK8 to the multivariate regression model with CSF Aβ_42_ as dependent variable and KLK8 as independent variable, with the covariates sex, age and the ApoE ε 4 allele frequency, the model resulted in an increased adjusted R² of 0.758 (p= 0.015) with β_KLK8_= -2.910 (p= 0.015), β_Ralb_= -1,040 (p= 0.116), β_IntKLK8xRalb_= 2,933 (p= 0.058), β_ApoE_= -0.520 (p= 0.015). In AD patients, the same regression model was not significant (adjust. R²= -0.197, p= 0.949). Including R_alb_ X KLK10 instead of R_alb_ X KLK8 did not result in a significant model neither in NC (p= 0.774) nor in AD (p= 0.686).

**4. Supplementary tables**

Table S1: Correlation of KLK6, KLK8 and KLK10 with AD biomarker in CSF in AD and NC

| **AD (n= 32)** | **CSF-tTau** | **CSF-pTau** | **CSF-Aβ42** |
| --- | --- | --- | --- |
| **Kolmogorow-Smirnow test** | p= 0.042 | p= 0.020 | p= 0.200 |
| **CSF-KLK6** | Spearman-Rho r= 0.600, p< 0.001 | Spearman-Rho r= 0.781, p< 0.001 | Pearson r= 0.212, p= 0.244 |
| **CSF-KLK8** | Spearman-Rho r= 0.175, p= 0.339 | Spearman-Rho r= 0.187, p= 0.305 | Pearson r= 0.072, p= 0.696 |
| **CSF-KLK10** | Spearman-Rho r= -0.095, p= 0.604 | Spearman-Rho r= -0.045, p= 0.807 | Pearson r= 0.337, p= 0.059 |

| **NC (n= 23)** | **tTau** | **pTau** | **Aβ42** |
| --- | --- | --- | --- |
| **Kolmogorow-Smirnow test** | p= 0.096 | p= 0.009 | p= 0.200 |
| **CSF-KLK6** | Pearson r= 0.397, p= 0.061 | Spearman-Rho r= 0.393, p= 0.064 | Pearson r= 0.119, p= 0.588 |
| **CSF-KLK8** | Pearson r= 0.202, p= 0.355 | Spearman-Rho r< 0.001, p= 0.998 | Pearson r= -0.478, p= 0.021 |
| **CSF-KLK10** | Pearson r= -0.080, p= 0.715 | Spearman-Rho r= 0.229, p= 0.294 | Pearson r= -0.062, p= 0.779 |

Legend to Table S1: Pearson correlation coefficients were described if variables were normally distributed (Whitney-U test > 0.05), otherwise Spearman-Rho was used. NC: cognitively normal controls, AD: patients with Alzheimer’s disease, CSF: cerebrospinal fluid.

Table S2

Associations of KLKs and clinical severity

| Group | Parameter | KLK6 | KLK8 | KLK10 |
| --- | --- | --- | --- | --- |
| AD | MMSE | r= -0.072  p= 0.702 | r=0.178  p= 0.339 | r= 0.292  p= 0.110 |
| AD | global CDR | r= -0.239  p= 0.187 | r= -0.051  p= 0.782 | r -0.232  p= 0.202 |
| NC | MMSE | r= -0.096  p= 0.663 | r= 0.214  p= 0.327 | r= 0.038  p= 0.864 |

Legend to Table S2: Spearman correlations among concentrations of KLK6, KLK8 and KLK10 in CSF with MMSE, global CDR in all patients (n= 32, n=31 for MMSE) or the NC (n=23). NC: cognitively normal controls; CDR: clinical dementia rating scale; MMSE: Mini-Mental state examination, KLK: kallikrein related peptidase, r: correlation coefficient.

Table S3

Univariate linear regression models with associations of PET-AD-biomarkers with KLK6, KLK8 and KLK10 in a sub cohort of 14 AD patients

| **Global PET signal ratios** | **CSF-KLK6**  **model statistics**  **(p_model,_ adjusted R²)** | **CSF-KLK8**  **model statistics**  **(p_model_, adjusted R²)** | **CSF-KLK10**  **model statistics**  **(p_model,_ adjusted R²)** |
| --- | --- | --- | --- |
| C/cerebellum-  [^18^F]FDG-PET signal | p=0.638  adj. R²=-0.063 | p=0.550  adj. R²=-0.050 | p=0.639  adj. R²=-0.063 |
| C/thalamus-  [^18^F]FDG-PET signal | p=0.534  adj. R²=-0.048 | p=0.632  adj. R²=-0.062 | p=0.069  adj. R²=0.187 |
| C/pons-  [^18^F^]^FDG-PET signal | p=0.167  adj. R²=0.187 | p=0.574  adj. R²=-0.054 | p=0.294  adj. R²=0.015 |
| C/vermis-  [^11^C]PiB-PET signal | p=0.573  adj. R²=-0.048 | p=0.508  adj. R²= -0.043 | p=0.678  adj. R²=-0.067 |

Legend to Table S3: linear regression models of different normalized global PET uptake signals as depended variable and each KLKs each as independent variable without covariables included. None of these analyses reached statistical significance (p< 0.05).

Table S4

1. Cluster distribution of the association of CSF-KLK6 with the cerebral amyloid controlled for ApoE ε4 allele frequency

| Cluster of 52321 voxels | % Cluster | % Label | Nb Vx Label |
| --- | --- | --- | --- |
| Temporal, right inferior | 4.67 | 68.71 | 3557 |
| Occipital, mid left | 3.46 | 55.35 | 3270 |
| Temporal, mid left | 3.36 | 35.35 | 4942 |
| Precentral, left | 3.34 | 49.63 | 3526 |
| Temporal, mid right | 3.19 | 37.90 | 4409 |
| Occipital, mid right | 2.77 | 69.11 | 2098 |
| Parietal, sup left | 2.66 | 67.41 | 2065 |
| Cerebelum Crus, left | 2.39 | 48.10 | 2603 |
| Precentral, left | 3.34 | 49.63 | 3526 |
| Precuneus, left | 2.33 | 36.92 | 3381 |
| Calcarine, left | 2.33 | 50.74 | 2300 |
| Precentral, right | 2.33 | 36.02 | 3381 |
| Lingual, right | 2.23 | 50.74 | 2300 |
| Parietal superior, right | 2.17 | 51.13 | 2222 |
| Calcarine, right | 2.14 | 60.24 | 1861 |
| Frontal, mid right | 2.09 | 22.51 | 4860 |
| Temporal, inferior left | 1.94 | 39.91 | 2518 |

1. Cluster distribution of the association of CSF-KLK6 with the cerebral glucose metabolism

| Cluster of 20840 voxels | % Cluster | % Label | Nb Vx Label |
| --- | --- | --- | --- |
| Occipital, mid left | 12.52 | 79.82 | 3270 |
| Temporal, mid left | 12.22 | 51.54 | 4942 |
| Temporal, inf left | 7.21 | 46.94 | 3200 |
| Fusiform, left | 7.03 | 63.42 | 2310 |
| Lingual, left | 5.18 | 51.50 | 2095 |
| Temporal, sup left | 4.44 | 40.29 | 2296 |
| Calcarine, left | 4.40 | 40.57 | 2258 |
| Precuneus, left | 4.27 | 25.23 | 3528 |
| Occipital, inf left | 4.02 | 89.05 | 941 |
| Angular, left | 3.61 | 64.19 | 1173 |
| Occipital, sup left | 3.22 | 49.19 | 1366 |
| Cuneus, left | 3.05 | 41.68 | 1526 |
| Parietal, inf left | 2.62 | 22.27 | 2447 |
| Parietal, supleft | 1.94 | 19.61 | 2065 |

1. Cluster distribution of the association of CSF-KLK10 with the cerebral glucose metabolism

| Cluster of 67504 voxels | % Cluster | % Label | Nb Vx Label |
| --- | --- | --- | --- |
| Lingual | 2.90 | 85.09 | 2300 |
| Temporal, sup right | 2.58 | 55.49 | 3141 |
| Occipital, mid left | 2.36 | 48.69 | 3270 |
| Fusiform, right | 2.24 | 60.17 | 2518 |
| Calcarine, right | 2.16 | 78.18 | 1861 |
| Occipital, mid right | 2.09 | 67.30 | 2098 |
| Temporal, mid right | 2.07 | 31.69 | 4409 |
| Cuneus, right | 1.89 | 89.75 | 1424 |
| Precentral, right | 1.88 | 37.62 | 3381 |
| Cingulate, mid right | 1.77 | 54.24 | 2203 |
| Occipital, sup right | 1.74 | 82.94 | 1413 |

Legend to Table S4: Supratentorial cluster distribution of the association of CSF-KLK6 with the cerebral amyloid controlled for ApoE ε4 allele frequency (A), as well as CSF-KLK6 (B) and CSF-KLK10 with the cerebral glucose metabolism (C). Global clusters are assigned to regions of interests (ROIs) and their relative percentage of the global cluster (% cluster), percentage of significant voxels in a ROI (% label) and absolute number of significant voxels in a ROI (Nb Vx). Cluster distribution was performed with SPM 12, automated anatomical labeling atlas of Tzourio-Mazoyer et al. (12)

**5. Supplementary figures**

Figure S1: Overlay of significant clusters of the associations of CSF-KLK6 with the FDG-PET and PiB-PET signal


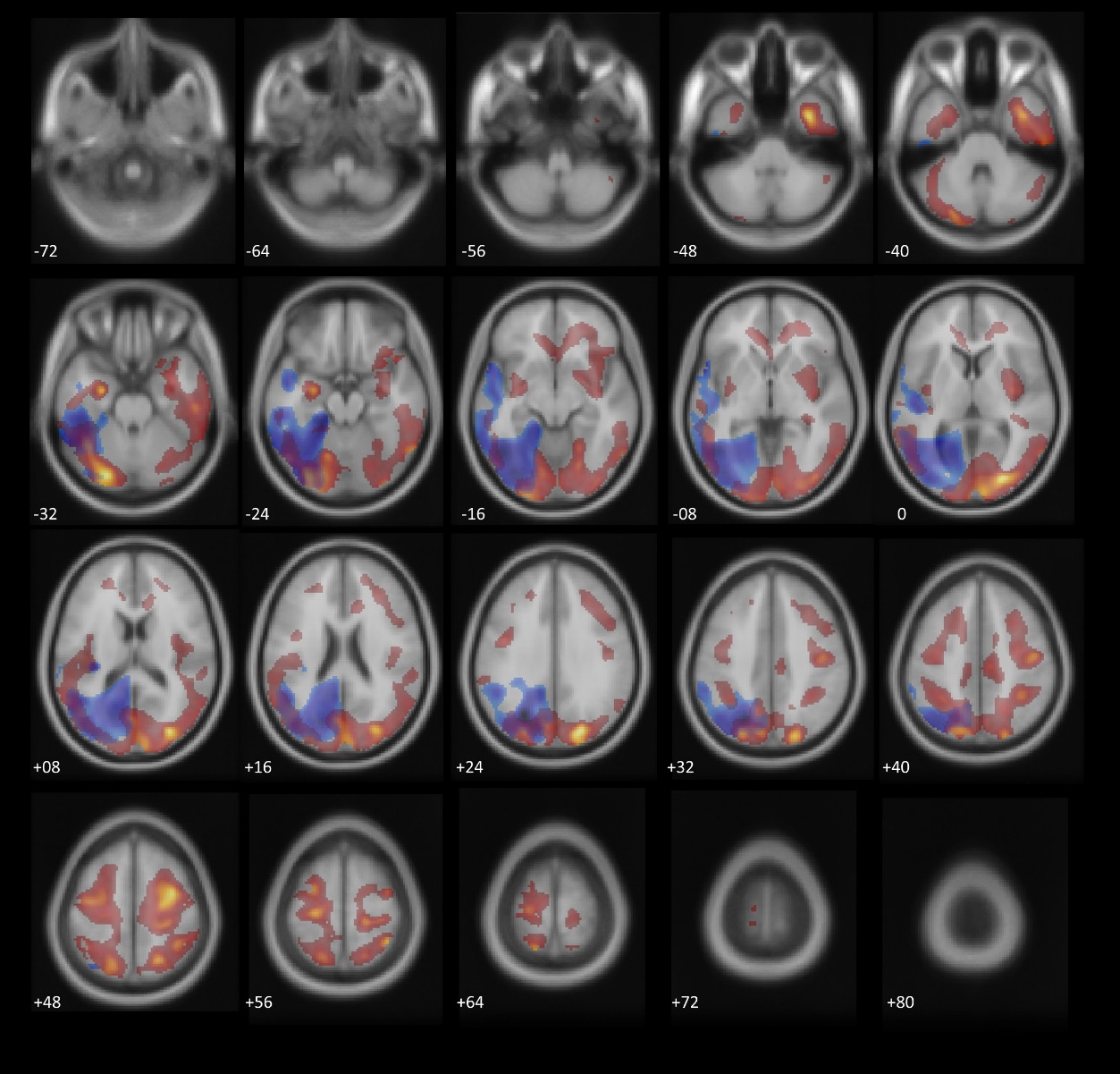


Legend to Figure S1: Overlay of significant (p<0.05 FDR corrected) clusters of voxel based regional regression analyses between CSF-KLK6 levels and the cerebral C/vPiB-PET signal (negative associations in red to yellow), controlled for copies of the ApoE ɛ4 allele and the C/pFDG-PET signal (positive associations in blue, respectively). Clusters are projected on axial T1 MRI scans (average of 152 scans, implemented in SPM12), numbers indicate z-coordinates of slices in Talairach space in mm. CSF: cerebrospinal fluid; KLK: kallikrein-related peptidase, ApoE ɛ4: apolipoprotein E epsilon 4; C/v: cerebrum to vermis; C/p: Cerebrum to pons; [C11]PiB-PET: [C11]Pittsburgh Compound B-positron emission tomography, [18F]FDG-PET: [18F]fluorodeoxyglucose-position emission tomography.

**6. Supplementary Material References**

1. Minoshima S, Frey KA, Koeppe RA, Foster NL, Kuhl DE. A diagnostic approach in Alzheimer’s disease using three-dimensional stereotactic surface projections of fluorine-18-FDG PET. J Nucl Med Off Publ Soc Nucl Med. 1995 Jul;36(7):1238–48.

2. Hoffman JM, Welsh-Bohmer KA, Hanson M, Crain B, Hulette C, Earl N, et al. FDG PET imaging in patients with pathologically verified dementia. J Nucl Med Off Publ Soc Nucl Med. 2000 Nov;41(11):1920–8.

3. Jagust W, Reed B, Mungas D, Ellis W, Decarli C. What does fluorodeoxyglucose PET imaging add to a clinical diagnosis of dementia? Neurology. 2007 Aug 28;69(9):871–7.

4. Grimmer T, Henriksen G, Wester HJ, Forstl H, Klunk WE, Mathis CA, et al. Clinical severity of Alzheimer’s disease is associated with PIB uptake in PET. Neurobiol Aging. 2009 Dec;30(12):1902–9.

5. Grimmer T, Riemenschneider M, Forstl H, Henriksen G, Klunk WE, Mathis CA, et al. Beta amyloid in Alzheimer’s disease: increased deposition in brain is reflected in reduced concentration in cerebrospinal fluid. Biol Psychiatry. 2009 Jun 1;65(11):927–34.

6. Grimmer T, Tholen S, Yousefi BH, Alexopoulos P, Forschler A, Forstl H, et al. Progression of cerebral amyloid load is associated with the apolipoprotein E epsilon4 genotype in Alzheimer’s disease. Biol Psychiatry. 2010 Nov 15;68(10):879–84.

7. Ishii K, Imamura T, Sasaki M, Yamaji S, Sakamoto S, Kitagaki H, et al. Regional cerebral glucose metabolism in dementia with Lewy bodies and Alzheimer’s disease. Neurology. 1998 Jul;51(1):125–30.

8. Waxman AD. H K.Lewis, DH.Herscovitch, P.Minoshima, S.Ichise, M.Drzezga, AE.Devous, MD.Mountz, JM. Society of Nuclear Medicine Procedure Guideline for FDG PET Brain Imaging. Proced Guid FDG-PET Brain Imaging. 2009 Society;1.0(1):1–12.

9. Grimmer T, Goldhardt O, Guo L-H, Yousefi BH, Förster S, Drzezga A, et al. LRP-1 polymorphism is associated with global and regional amyloid load in Alzheimer’s Disease in humans in-vivo. NeuroImage Clin. 2014;4:411–6.

10. Diamandis EP, Yousef GM, Soosaipillai AR, Grass L, Porter A, Little S, et al. Immunofluorometric assay of human kallikrein 6 (zyme/protease M/neurosin) and preliminary clinical applications. Clin Biochem. 2000 Jul;33(5):369–75.

11. Hegen H, Auer M, Zeileis A, Deisenhammer F. Upper reference limits for cerebrospinal fluid total protein and albumin quotient based on a large cohort of control patients: implications for increased clinical specificity. Clin Chem Lab Med. 2016 Feb;54(2):285–92.

12. Tzourio-Mazoyer N, Landeau B, Papathanassiou D, Crivello F, Etard O, Delcroix N, et al. Automated anatomical labeling of activations in SPM using a macroscopic anatomical parcellation of the MNI MRI single-subject brain. NeuroImage. 2002 Jan;15(1):273–89.
